# 628. The Dangers of Tabletop Burners

**DOI:** 10.1093/jbcr/irag033.454

**Published:** 2026-04-06

**Authors:** Tiffany N Lord, Elizabeth Allison Bowers, Joe Harvey

**Affiliations:** Evans-Haynes Burn Center at VCU Health, Richmond, VA; Evans-Haynes Burn Center at VCU Health, Richmond, VA; Chesterfield Fire and EMS, Chesterfield, VA

## Abstract

**Introduction:**

The U.S. Consumer Product Safety Commission reports there have been at least 60 injuries and two deaths since 2019, associated with tabletop burners. Tabletop burners are portable, ventless, devices that may be used indoors or outdoors. The devices contain a metal fuel box, where ethanol or alcohol-based fuel are added to create the flame. The use of ethanol/alcohol-based fuel creates a real flame, that is low heat and clean burning, with no smoke, soot or ash. As the fuel burns off, the flame can become non-visible. When the fuel box is refilled, while still ignited, or while the fuel box is still hot, there is a chance for flame jetting to occur. Flame jetting is a sudden explosive fire that occurs when the fire follows the fuel back into the container causing pressure to buildup then spraying the flaming fuel out of the container creating a flame thrower effect. Flame jetting can shoot up to 15 feet, engulfing bystanders and structures in flames. There are no current standards that require fueling containers to have a flame arrester, or flame mitigation device (FMD), which could prevent a flame from entering the container.

**Methods:**

A local fire agency identified similarities in two calls they received. Both calls were a result of the use of a tabletop device that resulted in burn injuries. To gain a better understanding, online research was completed, and a variety of devices and fuel were purchased, with the intent of re-creating flame jetting in a controlled setting. Two separate assessments of the devices occurred at a fire training center.

**Results:**

As the fuel levels become low in these devices it is very difficult to see the flame. The included instructions recommend that once the fuel becomes low, a lid should be placed on the container and the device should cool for 30-45 minutes prior to refueling and reigniting.

The fire agency was able to replicate flame jetting in one of the two validation trials. Documentation of the trails were sent to the Consumer Product Safety Commission for their review.

During the second testing, the local burn center was present, and the media was invited to observe and interview the content experts. The media team created a public outreach piece that was shared with the local communities and remains available on social media.

**Conclusions:**

Improper use of the device resulted in multiple incidents of significant injury and all tabletop fire devices should be banned for their risk of safety. There should be a safety requirement in place to ensure flame arrestors are placed on all fuel bottles sold to the public to minimize the incidence of flame jetting. Additionally, public outreach and awareness to educate the community on how to safely utilize the devices should be widespread.

**Applicability of Research to Practice:**

A product safety warning and recall was issued in December 2024, requesting that consumers immediately stop using and dispose of these products.

**Funding for the study:**

N/A.

